# Barriers in reaching new-borns and infants through home visits: A qualitative study using nexus planning framework

**DOI:** 10.3389/fpubh.2022.956422

**Published:** 2022-09-29

**Authors:** Vaishali Deshmukh, Shibu John, Abhijit Pakhare, Rajib Dasgupta, Ankur Joshi, Sanjay Chaturvedi, Kiran Goswami, Manoja Kumar Das, Rupak Mukhopadhyay, Rakesh Singh, Pradeep Shrivastava, Bhavna Dhingra, Steven Bingler, Bobbie Provosty Hill, Narendra K. Arora

**Affiliations:** ^1^The INCLEN Trust International, New Delhi, India; ^2^School of Management and Business Studies, Jamia Hamdard, New Delhi, India; ^3^Department of Community Medicine, All India Institute of Medical Sciences, Bhopal, Madhya Pradesh, India; ^4^Centre of Social Medicine and Community Health, Jawaharlal Nehru University, New Delhi, India; ^5^Department of Community Medicine, University College of Medical Sciences, New Delhi, India; ^6^Department of Community Medicine, All India Institute of Medical Sciences, New Delhi, India; ^7^Department of Pediatrics, All India Institute of Medical Sciences, Bhopal, Madhya Pradesh, India; ^8^Concordia–Architecture, Planning, Community Engagement, New Orleans, LA, United States

**Keywords:** home visitation, new-born care, community health worker (CHW), ASHAs (accredited social health activists), India, nexus planning

## Abstract

**Background:**

Home visitation has emerged as an effective model to provide high-quality care during pregnancy, childbirth, and post-natal period and improve the health outcomes of mother- new born dyad. This 360^0^ assessment documented the constraints faced by the community health workers (known as the Accredited Social Health Activists, ASHAs) to accomplish home visitation and deliver quality services in a poor-performing district and co-created the strategies to overcome these using a nexus planning approach.

**Methods:**

The study was conducted in the Raisen district of Madhya Pradesh, India. The grounded theory approach was applied for data collection and analysis using in-depth interviews, and focus group discussions with stakeholders representing from health system (including the ASHAs) and the community (rural population). A key group of diverse stakeholders were convened to utilize the nexus planning five domain framework (social-cultural, educational, organizational, economic, and physical) to prioritize the challenges and co-create solutions for improving the home visitation program performance and quality. The nexus framework provides a systemic lens for evaluating the success of the ASHAs home visitation program.

**Results:**

The societal (caste and economic discrimination), and personal (domestic responsibilities and cultural constraints of working in the village milieu) issues emerged as the key constraints for completing home visits. The programmatic gaps in imparting technical knowledge and skills, mentoring system, communication abilities, and unsatisfactory remuneration system were the other barriers to the credibility of the services. The nexus planning framework emphasized that each of the above factors/domains is intertwined and affects or depends on each other for home-based maternal and newborn care services delivered with quality through the ASHAs.

**Conclusion:**

The home visitation program services, quality and impact can be enhanced by addressing the social-cultural, organizational, educational, economic, and physical nexus domains with concurrent efforts for skill and confidence enhancement of the ASHAs and their credibility.

## Introduction

Home visitation is an effective and community-wide acceptable model to provide intensive care during pregnancy, childbirth, and postnatal care to improve maternal and child health outcomes (1, 2). Most of the home visitation programs are grounded on the ecological theory suggesting that the children develop in multi-layered environmental conditions (3). It links the parents-child-family and the health systems (4) and is a cost- and time-effective intervention (5, 6) that empowers skill, knowledge, and awareness of the mother on childcare, early identification of sickness, and care-seeking (7, 8), thereby improves the maternal and neonatal outcomes (9, 10).

ASHAs (Accredited Social Health Activists) are the community health functionaries stationed in the villages (1 ASHA per 1,000 population) and over a million ASHAs are working in 600000 villages across India (11). As part of the Janani Shishu Suraksha Karyakaram (JSSK, June 2011) (12) and Home Based Newborn Care (HBNC, August 2011) programs, ASHAs have been entrusted to visit pregnant women, facilitate institutional delivery, and make 6–7 home visits during the post-natal period to empower the mothers and mobilize the beneficiaries to health facilities, when required (13). Later in 2013, as part of the Norwegian India Partnership Initiative (NIPI) (14), the Home-Based New-born Care Plus (HBNC plus) program was piloted in four states of India (Rajasthan, Madhya Pradesh, Bihar, and Odisha). The HBNC Plus involved four more home visits for the infants after the postnatal period, at ages 3, 6, 9 and, 12 months to provide counseling for age-appropriate feeding, hand washing, regular play and, communication for early child care and development (ECCD), immunization, prophylactic oral rehydration solutions (ORS) and Iron and folic acid (IFA) distribution and growth monitoring.

Studies have shown that when an adequate number of home visits are made for newborns and infants accomplishments of the tasks assigned to community health workers lead to both better physical and cognitive development and early identification of sickness. High-quality home visitations also decreased the mortality and morbidities in this age group (15–17). An ASHA evaluation across seven Indian states demonstrated heterogeneity in-home visitation patterns both in terms of number and quality of visits (18). Mothers rated the performance of ASHAs as poor for advice/counseling regarding obstetric danger signs, neonatal assessment and, care in Karnataka, India (19). The internal program evaluation document of the NIPI program also showed that home visitations by ASHAs under HBNC and HBNC Plus programs were both inadequate and poor in quality and < 10% of sick young infants were mobilized by the frontline workers to health facilities (20). The barriers to timely home visitation and high-quality service delivery by the ASHAs are only partially understood. The social, cultural, and personal constraints have not been comprehensively researched in most of the studies, and the focus was generally limited to programmatic challenges (21). The HBNC Plus program evaluation highlighted the difficulty of translating the inputs, such as training and incentives into the desired outputs, such as home visits, both quantity- and quality-wise (20). The current study was therefore undertaken to document the barriers to completion of expected home visitation and quality of service delivery by the ASHAs as part of the HBNC (during the first six weeks after birth) and HBNC Plus programs (during the post-neonatal period through the first year of life) in Raisen district of Madhya Pradesh, India.

We applied the nexus planning framework (NPF), which is based on 360-degree assessment of the challenges, interlinkages, synergies and, trade between the challenges with emphasis on cross-sectoral sustainability and enhancing the socio-economic-cultural-and organizational resilience against current and future barriers. The NPF method was a further refinement of the traditional formative component recognizing the importance of sub-group and sub-constituencies of the stakeholders and attempts to incorporate their respective concerns, hesitancy and, fears and unique solutions (22).

## Methods

### Study site

The study was conducted in Raisen district of Madhya Pradesh, India from October 2016 to September 2018. Madhya Pradesh has one of the highest neonatal and infant mortality rates in the country and is one of the eight socio-economically backward states (Bihar, Chhattisgarh, Jharkhand, Orissa, Rajasthan, Uttaranchal and, Uttar Pradesh), also referred to as the Empowered Action Group (EAG) states (23). In Madhya Pradesh, HBNC Plus pilot program was rolled out by NIPI in four districts (out of 51 districts) namely Raisen, Hoshangabad, Betul and, Narsinghpur. These districts are not part of the 123 aspirational districts declared by NITI Aayog but their overall rankings for development including health indicators are in the lower one-third of all districts of the country (24). Raisen district had poor indicators for mobilization of home- delivered mother-child dyad to skilled health personnel within 48 h, and dispensing of ORS to diarrhea patients among the four NIPI districts (25, 26). The Department of Health and Family Welfare, Government of Madhya Pradesh, NIPI leadership and, the investigators jointly decided to include Raisen district for the evaluation of ASHA performance for home visitations under HBNC and HBNC Plus programs. In the district, four administrative blocks were purposively selected for the study i.e., Gairat Ganj (53 kms), Begam Ganj (78 kms), Silwani (86 kms), and Udaipur (80 kms), based on their distance from the district headquarter.

### Study methods

The study had two phases data collection, analysis and, synthesis. During Phase I (formative research), we applied the qualitative research methods using grounded theory for data collection and analysis. In-depth interviews (IDIs) and focus group discussions (FGDs) with various participants were conducted at their respective locations. Phase-II: Data from Phase-I were used for conducting the multi-stakeholder nexus planning (NP) exercise.

### Phase I: Formative research

#### Participant selection

**Figure 1:**
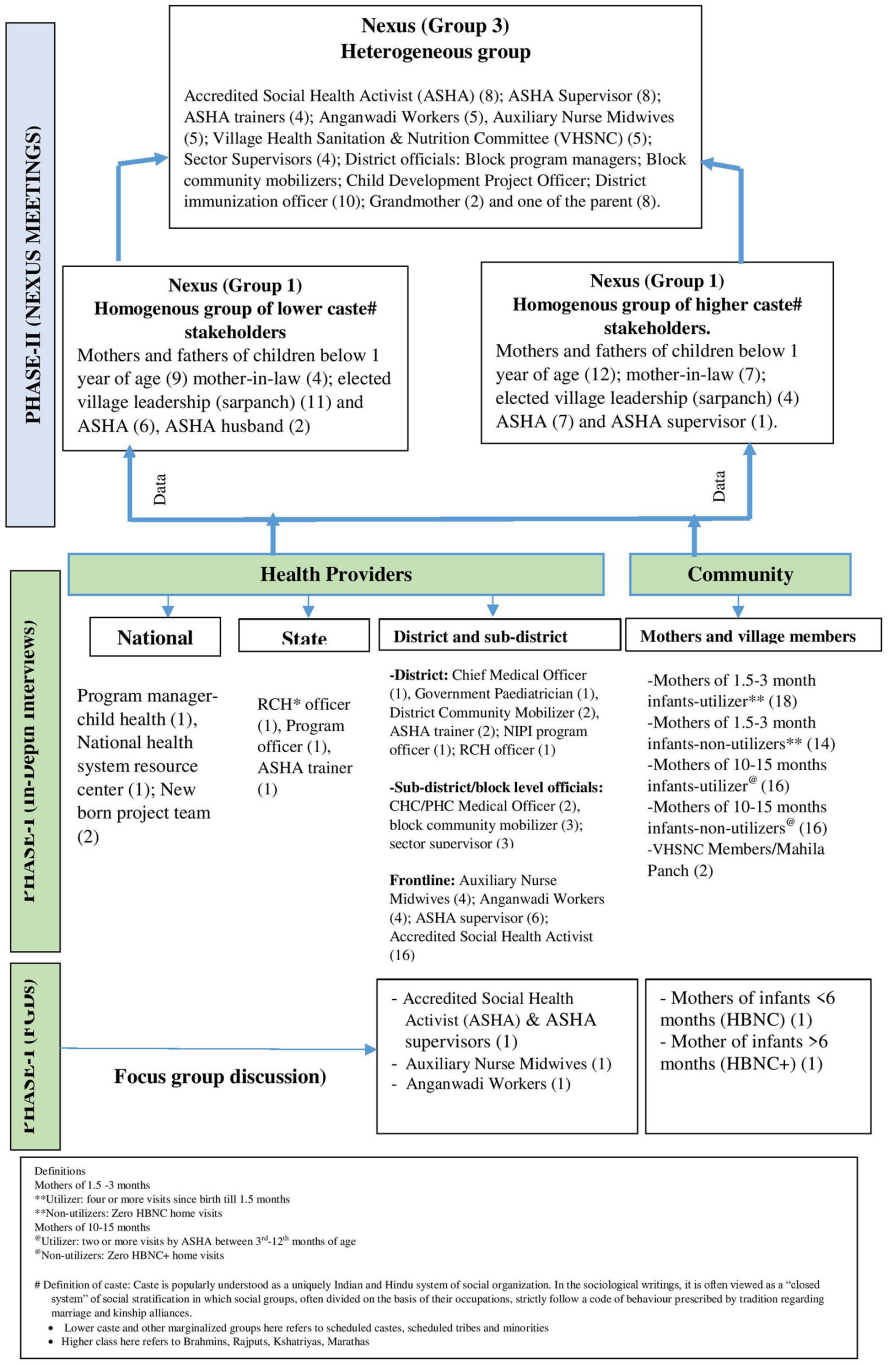
Stakeholders involved in the study.

Community engagement: Before commencing our community activities, we had a series of meetings with village leaderships (Panchayat members) in the four study blocks to explain to them the purpose and methods of the study and obtain their buy-in. We purposively selected the respondents for IDIs and FGDs ensuring the representativeness at different levels (national, state, district, and community levels) (Figure 1). We purposively selected the respondents from different stakeholder categories (home visitation beneficiaries, ASHAs, and other frontline health and Anganwadi workers residing and working in these four blocks, healthcare providers and, program managers working at block, district, state and, national levels). At this stage, the caste of the stakeholder (lower/backward and upper/forward) was neither considered for the selection nor was not recorded to avoid any bias. The investigator group decided following definitions to define beneficiary mothers as utilizers of the home visitation by ASHA: (a) under HBNC program: 4 or more home visits done during 1.5 months after birth; (b) under HBNC Plus program: 2 or more home visits done between 3 and 12 months of age. Non-utilizers had not received any home visits. The quality of each home visit was assessed with reference to the program guidelines (13, 27). A prior appointment was taken with the interviewee before all interactions and during the face-to-face interaction.

#### Data collection and analysis

We collected data using pretested semi-structured IDI and FGD guides developed by the investigators/authors, a multi-disciplinary team. A team of four female social scientists trained in qualitative data collection conducted the IDIs and FGDs at places convenient to the interviewee and no observer was allowed. The IDIs and FGDs were conducted in the local language (Hindi) and audio recorded with consent. Some of the IDIs with health managers at the state and national level were in English; these were also recorded. Study investigators (VD, AP) conducted the IDIs at the national, state and district levels and facilitated the FGDs. Senior researchers (NKA, VD, AP, and SJ) with at least 15 years of experience in qualitative research, supervised data collection, and analysis. Most of the IDIs and FGDs lasted for about one hour (range 50 to 120 min).

All IDIs and FGDs were transcribed (i.e., Hindi) and then translated into English. The transcriptions/translations of all IDIs and FGDs were entered using INCLEN qualitative data analysis software (IQDAS) (28) for systematic organization and retrieval of data for analysis. During quality assessments, four IDIs were rejected due to incompleteness (one) and poor quality audio (three). We adopted the grounded theory (29) approach for data analysis. All the IDI and FGD transcripts were carefully read by two trained social scientists and reconfirmed by a senior author (VD), to interpret the data. The researchers inductively analyzed the data using the following steps: (i) free-listing and open-coding of the responses and marking the relevant/introductory statements for use as quotable quotes; (ii) axial coding of the responses keeping in mind the connection/relationship between free-listed responses/small units, and (iii) develop selective codes (wherever applicable) for the clusters of axial codes communicating common theme. The emerging patterns/themes from the responses, helped the researchers to interpret the ASHA's functioning in the light of the different challenges. The emerging codes and themes were regularly discussed with the senior author (NKA) to resolve inconsistencies. All analytic steps were followed (open, axial, and selective coding) consistently to have inter-coder reliability and stability, and reproducibility (30). Data quality was ensured by triangulating data collection methods at two levels-across methods and across respondents (trustworthiness). The findings were expressed semi-quantitatively using qualifiers to express findings systematically: very few (< 10%), some (10–24%), about half (25–49%), the majority (50–75%), most (76–89%), and almost all (>90%). The quasi-quantitative expressions are based on how many times the code was mentioned (28, 31).

### Phase II: Nexus planning approach

#### Nexus planning framework (NPF)

**Figure 2:**
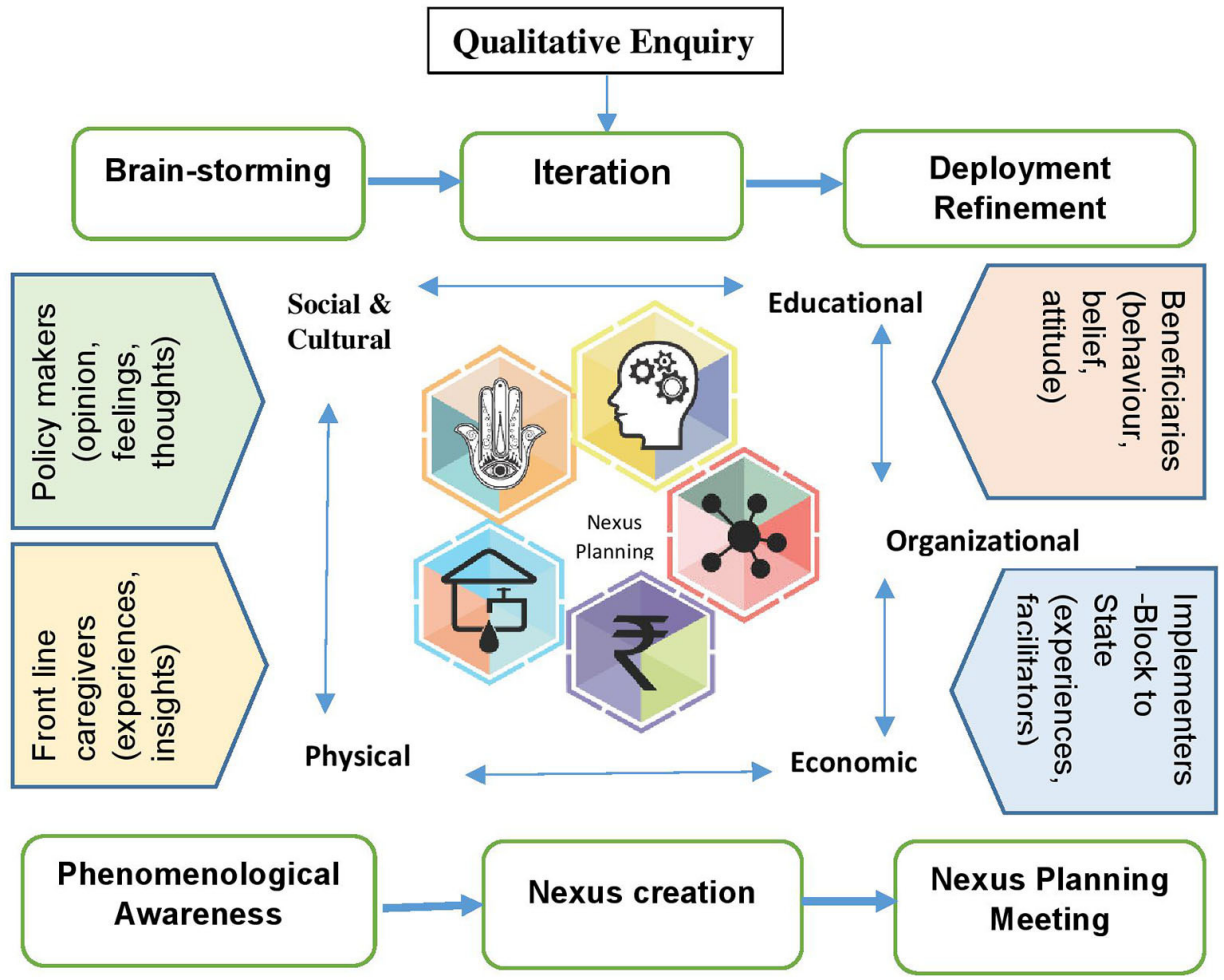
Nexus framework.

Nexus approach is currently used in a large number of sectors to define solutions to complex, multidimensional, and layered challenges. Under the nexus approach, interlinkages, synergies, and trade-offs are analyzed, with the aim of identifying solutions and improving the performance of ecosystems. Thus, nexus planning emphasizes cross-sectoral sustainability and enhances socio-economic-cultural-organizational resilience against current and future challenges. We have used NPF as a participatory co-creation process to ensure ownership of all stakeholders including their sub-constituencies in decision-making and bringing about accord among all stakeholders including the program implementers to improve the performance of ASHAs, particularly for home visitations under HBNC and HBNC Plus program. NPF systematically focuses on the challenges and attempts to identify the possible solutions under five nexus domains: social-cultural, economic, physical, organizational, and educational (22, 32–34) (Figure 2).

#### Process of nexus planning approach

The nexus planning process had three steps after the initial formative research component using qualitative methods (Phase I), which identified the barriers and facilitators to the home visitations by the ASHAs from a large number of stakeholders including the mothers and ASHAs (Figure 1). The data from Phase-I were analyzed and organized according to the five nexus domains for sharing with different stakeholders.

#### Nexus planning step-1

An extensive discussion was held between the investigators, field staff, and Panchayat members to identify essential sub-groups/constituencies of all stakeholders, which had different perspectives and social dynamics at the ground level. Through the discussion, then it was decided to conduct three nexus meetings/consultations considering the caste groups of the community and the frontline workers and larger community of service providers; one each including the representatives from lower and higher/upper castes (as per the local knowledge) and the third with mixed representatives drawn from all caste groups.

#### Nexus planning step-2

Each of the caste-specific nexus planning meetings/ consultation had 20–30 participants drawn from the same caste group (community and parents from all the four blocks, ASHAs from the respective blocks and two program managers from the district). The meetings were facilitated by the authors. The objectives of these nexus planning meetings/consultations were to discuss the findings from Phase-I (formative research); validate the sub-group specific observations emerging from the data analysis; identify the challenges categorized according to the nexus domains and suggest solutions appropriate for their respective caste groups.

#### Nexus planning step-3

Following the caste-specific nexus planning meetings/consultations, the final larger heterogeneous stakeholder group consultation (59 participants) was organized to bring synergy between the perspectives of the two sub-groups, smoothen the points of disagreement and develop final recommendations for improving the performance of the ASHAs in the communities. This nexus planning method is a further refinement of traditional formative components recognizing the importance of sub-group and sub-constituencies of the stakeholders, bringing about synergies, accord and identifying the points of negotiations so that the recommendations incorporate their respective concerns, hesitancy and fears, and unique solutions.

### Structure of nexus planning meetings (NPMs)

The nexus planning process was facilitated by two experts, who are the innovators of the process (BH, SB) and authors having experience in community engagement. The participants for nexus planning meetings included: (i) community stakeholders engaged in the process of home visitation directly or indirectly i.e., mothers, family members, village leadership (religious, social, and cultural); (ii) elected village leadership (Panchayat - local self-Government), Village Health, Sanitation, and Nutrition committee (VHSNC) members; (iii) representatives from education, revenue, and administration departments; (iv) respondents from the health department– ASHAs, ANMs, doctors from PHC/CHC and Chief Medical Officer of the district, ASHA supervisor; and (v) department of women and child welfare-AWWs. Three NPMs were organized: two preliminary NPMs considering the social demographic sensitivity [the community stakeholders were purposely drawn from low (first NPM) and high castes (second NPM)] and the third with a mix of community and representatives from the health system (Figure 1). No such differentiation was done for the healthcare providers and frontline workers for these NPMs.

First (32 participants; community members drawn from 15 villages) and second (31 participants; community members drawn from 8 villages) NPMs reviewed the findings of the IDIs and FGDs and indicated their views about the correctness of the data and its interpretation. The rationale of giving importance and hearing to all the sub-group stakeholders/constituencies brought out items and matters which required joint discussion to arrive at a mutually acceptable way forward. The challenges faced by ASHAs from forward and backward communities were different and had to be handled in a manner that required suggestions from both sides.

**Figure 3:**
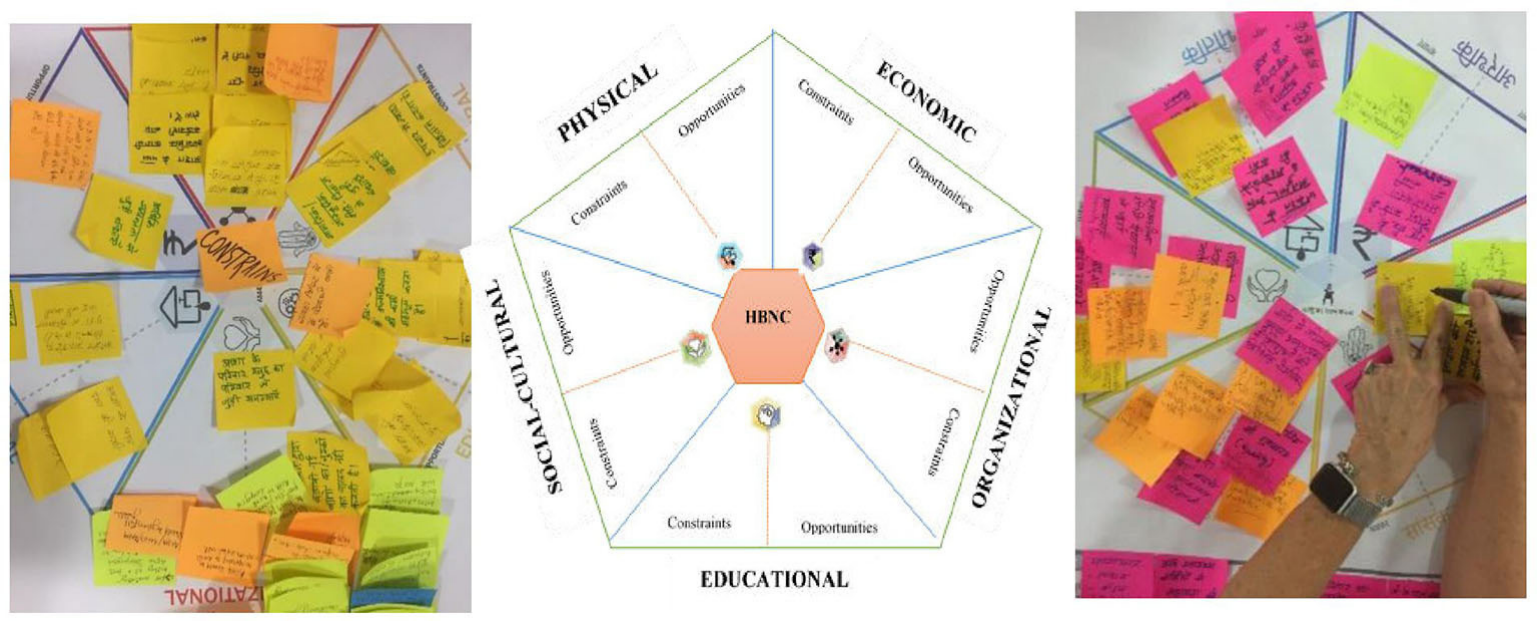
Nexus chart.

The third NPM (59 participants; community members from 22 villages) discussed the barriers and strategies for overcoming these in a harmonized, negotiated, and comprehensive manner. Each NPM had a similar work process. The objective of the initial plenary of 60 min, was to apprise the heterogeneous audience of the perceptions of the diverse sub-group stakeholders and emerging themes that required careful attention. The group was divided into 3 smaller groups and each comprised mix of community sub-groups/constituencies and geographies of the district along with health providers. Nexus planning charts were used primarily to keep the discussion on track document discussions according to specific domains, and develop final recommendations for improving the performance of the ASHAs in actual field settings (Figure 3). Finally, the plenary session of 90 min summarized the findings from all the sub-groups. The total duration of these meetings was approximately 3.5 to 4 h (including conduction). During the final plenary session, the three groups came together to build a consensus about the barriers and solutions and organized into the NPF. The summary also identified action for different stakeholders to improve the frequency and quality of home visitation and sustain these during the immediate post-partum period (HBNC period) and up to 15 months of age (HBNC Plus period).

#### Collation and interpretation of NPMs

The NPM facilitators noted down the observations and comments of every participant on ‘post-it/sticky notes' and collected these on the nexus domain chart. The findings from the first and second NPMs were shared with 3rd large group NPMs. The final plenary of the 3rd NPM helped in the co-identification of the barriers, and co-creation of the solutions and recommendations under the five nexus domains i.e., socio-cultural, physical, organizational, economic, and education.

## Results

### Phase I (qualitative data)

Figure 1 and Table 1 provide the distribution of stakeholders, and demographic profile of mothers and frontline workers participating in Phase-I of the study. A total of 119 IDIs were conducted: mothers (64), ASHAs (16), Auxiliary Nurse Midwives (ANMs) (4), Aganwadi workers (AWW) (4), ASHA supervisors (ASHA Sahyogini) (6), VHSNC members (2) and health system representatives at block, district, state and national levels (23). We also conducted five FGDs: one each with mothers of an infant under < 6 months old infants (for HBNC) and one with mothers of an infant 6 months to 15 months for (HBNC Plus program); one with ASHAs and Asha supervisors; one with ANMs and one with AWWs to supplement the IDIs for data saturation and data triangulation (Figure 1).

**Table 1:** The socio-demographic characteristics of the study participants.

**Mothers of children**	**HBNC**		**HBNC Plus**	
	**Mother utilizer [6 weeks-3months children] (*N* = 18)** **(%)**	**Mother utilizer [10-15 months child]** **(*N* = 16)** **(%)**	**Mother non-utilizer [6 weeks−3months child] (*N* = 14)** **(%)**	**Mother non-utilizer [10–15 months child] (*N* = 16)** **(%)**
**Literacy status of the mothers**				
Illiterate	3 (16.7)	7 (43.7)	2 (14.3)	4 (25)
< 5th standard	2 (11.1)	5 (31)	2 (14.3)	4 (25)
6–12 standard	12 (66.6)	4 (25)	10 (71.4)	8 (50)
>12 standard	1 (5.5)			
**Mother's occupation**				
Housewife	12 (66.6)	12 (75)	12 (85.7)	10 (62.5)
Working outside	6 (34.2)	4 (25)	2 (14.3)	6 (37.5)
**Literacy status of the fathers**				
Illiterate	3 (16.6)	2 (12.5)	2 (14.3)	3 (18.7)
< 5th standard	2 (11.1)	4 (25)	-	2 (12.5)
6–12 standard	12 (66.6)	10 (62.5)	11 (78.5)	11 (68.7)
>12 standard	1 (5.5)		2 (14.3)	
**Father's occupation**				
Farming (own farm)	4 (22.2)	6 (37.5)	5 (35.7)	8 (50)
Farming (laborer)	10 (55.5)	7 (43.7)	7 (50)	8 (50)
Self-employed (Shopkeeper/tailor/carpenter)	4 (22.3)	3 (18.8)	1 (7.14)	-
Government job	-	-	1 (7.14)	-
**Type of family**				
Extended	13 (72.2)	8 (50)	8 (57.1)	6 (37.5)
Joint	2 (11.1)	5 (31.2)	1 (7.1)	6 (37.5)
Nuclear	3 (16.7)	3 (18.7)	5 (35.7)	4 (25)
Religion (Hindu)	18 (100)	16 (100)	14 (100)	16 (100)
Frontline health functionaries	ANM^*^ (*N* = 4) %	AWW^*^ (*N* = 4) %	ASHA^*^ (*N* = 16) %	ASHA Supervisor (*N* = 6) %
**Age**				
< 30 years	-	-	6 (37.5)	3 (50)
31–40 years	1 (25)	2 (50)	6 (37.5)	3 (50)
>40 years	3 (75)	2 (50)	4 (25)	
Marital status (Married)	4 (100)	4 (100)	16 (100)	6 (100)
**Education**				
< 10th Standard	-	-	11 (68.8)	
10–12th standard	1 (25)	3 (75)	4 (25)	3 (50)
>Graduate	3 (75)	1 (25)	1 (6.3)	3(50)
**Years of working in current position in sub-centre/area**				
< 5 years	-	-	4 (25)	6 (100)
5–10 years	-	1 (25)	12 (75)	-
>10 years	4 (100)	3 (75)	-	-
Focus group discussion**s** (FGD) (*N* = 5)	ASHA^*^ (8) (1-FGD)	ANM^*^ (8) (1-FGD)	ASHA^*^ Supervisors (8) (1-FGD)	Mothers of infants (10+10) (2-FGD)
Age (in years)	25–40	30–40	30–40	20–32
Working in current position in sub-centre/area (in years)	5–10	10–12	5–10	-

* Accredited Social Health Activist (ASHA);

* AWW: Anganwadi Worker;

* Auxiliary Nurse Midwives (ANMs).

Data from Phase-I has been organized under the nexus domains with the emerging themes and sub-themes along with an illustrative statement from the participants.

### Social and cultural factors

#### Theme-1 caste, economic divide, and religious beliefs

The majority of the state and district level stakeholders, ANMs, and approximately half of the ASHAs identified socio-economic divide and caste/religion-based discrimination making the home visitations difficult and the reluctance of families to accept the health and wellness messages for childcare and more so for male children. Socio-cultural beliefs such as seclusion of post-partum mother and child and belief in the tradition of the‘evil eye effect' also made it difficult for ASHAs to deliver the desired services.

“*Some refuse to put the child in the weighing bag. They say that you took the weight of the Muslim child, the Scheduled tribes (ST) Child and now you will put our child in the same bag” [ASHA Sahyogini]*

“*She is from a rich family. Why will she will come to a low-income family house?” [Mother non-utilizers]*

“*Some are Harijan (Scheduled caste), and some are Vasur (scheduled caste)?, so she differentiates. She is not comfortable coming to our house. She does not touch our child.” [Mother non-utilizers]*

#### Theme-2 credibility of ASHA

Stakeholders from the administrative hierarchy and community perceived trust deficit and lack of respect and credibility of ASHA due to lower education status, inadequate training, and lack of confidence demonstrated while communicating with the family members. Similarly, the community questioned the ASHA's credibility, when the family experienced ‘inadequate or suboptimal' services at the health facility to which ASHA referred them. Several elders in the family preferred traditional childcare methods over ASHA's suggestions. Common superstitious beliefs such as ‘Sutak' (prohibitions observed during post-partum period), when the mother-neonate are confined indoors, were used as an excuse to refuse both contact/examination by the ASHA and referral. The front-line workers (ANMs, AWWs, and ASHA supervisors) perceived that it was difficult for ASHAs to convince the community and families about immunization and the associated minor side effects. Families also showed deep-rooted resistance in following advice, such as play and communication with the child and washing hands with soap, which considerably differed from their existing practices and were perceived as irrelevant to them. Approximately half of the non-utilizers mothers complained of the lack of resources required to follow ASHAs' advice. We also found a better understanding of utiliser mothers' knowledge of program components like exclusive breastfeeding, IFA syrup, hand washing, and ORS preparation when compared with the non-utilizers mothers.

“*People doubt her motive when she visits their house again and again”. [AWW]*

“*ASHA instructs mother what all things she should follow after delivery. Family members get irritated that why she is instructing her”. [ASHA Sahyogini]*

“*We don't expect much from ASHA since she doesn't know much herself.” [Mother, hard to reach]*

“*If ASHA asks them for vaccination and child has a mild fever, then they immediately say no. Few people don't behave well with the ASHA.” [ANM]*

#### Theme-3 personal factors and client family-related challenges of ASHA

The majority of the ASHAs felt that they were caught between their domestic (looking after children and husbands, household chores) and professional responsibilities. According to the ASHAs, the non-availability of a mother at the time of the home visit or the mother's engagements with social responsibilities posed additional challenges. Mothers were unhappy that most ASHAs did not maintain a timetable for upcoming home visits or were informed of a home visit beforehand. Many mothers went back to their natal homes after childbirth for 6–12 weeks. As a result, either the HBNC visits remained incomplete, or HBNC Plus visits began late.

“*I was having an issue with my mother-in-law because she repeatedly kept asking where I went. I have to tell her that I have to measure weight by going house to house as an ASHA responsibility. Then she allows me to go.” [ASHA]*

“*Other issues are mother goes to her maternal place along with child or if the child is underweight then she is referred to hospital and sometime ASHA is herself tied up with some work in such cases she is not available to make home visits.” [ASHA Sahyogini]*

### Educational factors

#### Theme-4 capacity building

The health supervisors expressed concern about the ASHA training including residential training (staying away from the residence), curriculum design and its poor translation; and organization of practical skill training. However, most ASHAs and ASHA supervisors did not point out any difficulty in attending residential training. ASHAs and their supervisors reported that the training was insufficient and unsuitable; difficulty in comprehending technical terms related to many health and medical science by the ASHAs with less formal education, the content of the curriculum material, and the pace of knowledge imparting procedures in real-life situations.

“*As and when we come back home, then we cannot learn further anything under the supervision and become busy with village and family chores. And after some time, many forget their training.” [ASHA]*

“*Personally I feel ASHA is not a proper person to identify neonatal sepsis. We have trained we have to define the criteria for what are the danger signs and how to identify them. I think it is difficult for women from the field with a limited knowledge.” [State Officer]*

“*ASHAs hardly retain about 50% of training.” [ASHA Sahyogini]*

#### Theme-5 communication and technical skills

One of the most critical determinants of ASHA's credibility and acceptability in the community was its technical competence and communication skills. The ASHAs' inability to recognize sickness in children and the rationale and basis of nutritional and play therapy made it challenging to communicate messages correctly, effectively, and confidently. State and district level stakeholders acknowledged the poor quality of home visits because of the ASHAs' technical and communication competence challenges despite frequent contact. Although half of the ASHAs responded that they don't experience any difficulty performing HBNC/HBNC Plus responsibilities, almost a similar proportion accepted that they encountered problems while making home visits. ASHAs acknowledged that some of the poor response from mothers and family members was on account of their inability to communicate clearly and effectively. Some ASHAs reported having difficulty in performing technical tasks such as measuring temperature, weighing new-born, and making growth charts.

“*I don't have enough knowledge to know whether he has a fever or not. We make an idea by seeing whether he is playing or not. [ASHA]*

“*She is not able to explain when and how much IFA syrup is to be given and why it should be given”. [Block Medical Officer]*

“*ASHAs hardly retain about 50% of training.” (Echoed by many ASHA Sahyoginis)”*

“*She is unable to form connectivity. When she goes for a home visit, she talks about general household things such as cooking and does not discuss health services. They forget total about health and wellness issues. It is the main gap.” [District Level ASHA Trainer]*

### Organizational issues

#### Theme-6 supportive supervision

ASHA supervisors claimed that they monitored and supported the ASHA's activities. State and regional managers, however, acknowledged that the supervision of ASHAs was both poor and unsatisfactory because of vacancies in the supervisory cadre (ASHA Supervisors). The HBNC/HBNC Plus performance formats/reports were reviewed infrequently and supervision was of limited utility. The program managers strongly contended that state and district-level officers should also visit the field to understand and address the managerial bottlenecks. Block and community-level stakeholders mentioned the availability of a limited mobility budget for monitoring was also a hurdle in program monitoring.

“*Supportive Supervision is one of the weakest links in-home visitation, which was the strongest in the Abhay Bang Gadchiroli project.” [National level stakeholder]*.

“*At the block level, we have to check the filled forms; we are unable to check it properly due to a high number of forms being submitted at the block level every month. It becomes difficult to review all the forms properly.” [Block community mobilizer]*

#### Theme-7 planning and management of field activities

ASHAs, their supervisors, and senior officers consistently accepted that the tasks assigned to ASHAs were complex. The ASHAs were imparted limited exposure to planning and prioritization exercises during training. Not infrequently, ASHAs re-prioritized their work based on directives from higher-ups for health and non-health exigencies beyond the planned activities. Vacancies and rapid turnover among ASHAs and vacant positions in supervisory cadres added to poor planning and management.

“*ASHA has many other responsibilities, prioritization and non-adherence to the schedule should be seen in that context.” (Echoed by many ASHA Sahyoginis)*

“*There are 5-10 percent vacancies for the post of ASHA Sahyogini, ASHA, and BM (block mobilizer)” [Programme Officer]*

#### Theme-8 availability and quality of supplies

Irregular and insufficient supply of products (such as ORS and IFA syrup) and stationery adversely affected the HBNC/HBNC Plus related functioning of the ASHAs. The managers attributed this to distribution process-related challenges and delays by ASHAs to indent the supplies.

“*Last year in January, my BP and weighing machine were not working. So I deposited in last February, and I got this February.” [ASHA]*

### Economic issues

#### Theme-9 incentives and availability of resources

ASHAs are remunerated on the basis of the completed tasks (such as vaccination or home visits). Most ASHAs complained about the delay in receiving the incentives. The factors identified for the delays included: late receipt of funds, inability to fill the records appropriately, and refusal by the families to comply with their advice regarding immunization and sickness and hospital referrals. On the other hand, supervisors pointed to the challenges in releasing the incentives due to incorrect and incomplete maintenance of records and mismatch between claims and feedback from the field; shortage of funds was infrequent and not considered as significant concern. While most of the ASHAs and ASHA Supervisors reported receipt of only 50 percent to 70 percent of their entitled monthly incentives, the managers at the district level didn't agree with it and claimed that the full amount was distributed. The family members of ASHAs were reportedly unhappy about the lesser amount of incentives, delay in payments, and being paid less than other frontline workers (e.g., Anganwadi workers); which pose challenge in obtaining adequate support from their own family itself to perform the duties.

“*We get money after 2 or 3 months, and it's not monthly. Everyone gets after this much time. When we say this to sir, he says that I am the only person managing it, so delay happens, and there is no such work for which we should pay early.” [ASHA]*

### Physical issues

#### Theme 10 – ASHA's mobility within her village jurisdiction

The ASHAs and several program managers at different levels acknowledged the hurdles related to reaching outlying and hamlets, tribal areas, and areas with hilly terrain. Few of the ASHAs were not from the same village communities (though this is an essential mandate) and therefore needed to travel to their place of work.

“*Tribal population is living in far off areas. So need a strategy for better reach”. [NIPI Programme Officer]*

“*Some villages are too big, and houses are scattered. The area might be split into smaller ASHA jurisdictions.” [PHC Medical Officer]*

### Nexus planning meetings (NPM)

The details of the discussion held during the preliminary two caste-specific NPMs are presented in Table 2. The purpose of the NPMs was to identify the strategies to negotiate these challenges of the constructs by each of the social groups and help ASHAs better accomplish their other tasks in their community settings. The emerging themes and potential solutions were noted for both caste sub-groups. Most of the obstacles and the suggested approaches to overcome were similar across the groups, the lower and higher caste groups. The lower caste groups came up with suggestions to mitigate the caste and religion-related biases through awareness generation about the purpose of ASHAs' home visitations. Awareness regarding the value of home visitation and the role of ASHAs were considered essential for men from all communities, elder women who are particularly prejudiced, and the village leadership. The higher caste groups made additional recommendations on earning respect for ASHA by imparting her technical competence and good communication skills that would facilitate acceptance and performance. Through these collective efforts and discussions, we synthesized a broad range of views echoed by participants of the NPMs. The recommendations facilitated building contextual ownership and finding solutions to the barriers in making home visitation by the ASHAs.

**Table 2:** Preliminary nexus planning meetings with stakeholders (community, families, parents, ASHAs) separately with the lower caste (LC) and higher caste (HC) groups: summary of issues influencing home visitation by the ASHAs under HBNC & HBNC Plus programs.

**Domains**	**Domain specific challenges**	**LC**	**HC**	**Broad potential solutions**	**LC**	**HC**
**Social and cultural**						
Caste, economic divide & religious beliefs	Challenges due to cultural rituals/ social caste & religion conflicts /Social caste and religion based challenges	√	√	Men of the household must be involved more in negotiating such barriers.	√	X
	Higher caste families don't allow ASHAs to handle their child/ low caste ASHAs discriminated, not allowed into upper caste house	√	√	There should be multi-caste and religion meetings to discuss such issues in addition to the participation by the Panchayat.	√	X
	Differentiation between different social & religious classes / no acceptance of food and water from lower caste/poor	X	√	Similar caste/community should hold meetings and find ways to negotiate such barriers with members of the other community.	√	X
	Weighing babies from different castes and religion on the same weighing bag- makes it difficult for ASHAs to do her tasks	√	X	Panchayat should encourage higher caste ASHAs to visit all houses without discrimination	X	**√**
	Caste discrimination by women is more common compared to men of the same caste	√	√	Involve Panchayat in helping ASHAs overcome caste barrier	X	**√**
	Elder women (e.g., Mothers in law, grandmothers) discriminate more as compared to younger women	X	√	Higher designation of ANM makes her immune to caste discrimination; similarly ASHAs can be accorded higher official status	X	**√**
	During menstrual period women are not allowed to touch babies	√	√	Local women's group can help and explain to the families from all social sections to facilitate home visits.	X	**√**
	Social beliefs regarding restricted contact with recently delivered women and their babies	X	√	Panchayat members and Sarpanch can help ASHAs in visiting the households that are resisting and are a challenge	X	**√**
	Families trust traditional home remedies over ASHAs' advice / families believed in traditional home remedies	√	√	Families encouraged to adopt good traditional practices along with ASHAs advice for child's sickness and wellness	X	**√**
	Pressure from same community and clan members to maintain social and religious discrimination	X	√	To reduce the cast and social barriers, there is need for demonstration from the top political and administrative levels	X	**√**
	Some high caste women prefer home deliveries due to their superiority complex	X	√	Awareness about the high value for institutional deliveries for reluctant families	X	**√**
Personal barriers of ASHAs client family related challenges	Lack of support from ASHA's family - her husband, in-laws and other family members to work in their villages for health related issues	√	√	All family members need to be oriented on the ASHAs tasks, its importance to community health and need of home visitations for the wellbeing of both the mothers and their babies	√	X
	Time constraint and pre-occupation with their household works, looking after children, husband, family members and cattle	√	√	If incentive is received in timely manner, and ASHAs are considered as contributing to the family resources, getting the support from ASHAs' family members including need to go out during odd hours shall be easier	√	X
				Mothers in law and other members help ASHAs to share responsibilities in the household chores	X	√
				Panchayat members and Sarpanch can discuss the problem with the male members and elders of the families who are interfering the ASHAs activities vis-à-vis their need at household front	√	√
	Adverse social comments about ASHAs' work from their co-villagers and neighbors	X	√			
**Credibility**	Family & community do not have faith in ASHAs' activities and home visitations	√	√	ANM/MO and block level personnel use the same and endorse the messages and activities of ASHAs to the families and community in groups	X	√
				ASHAs respond to the calls and requests from households and mothers at all times	X	√
				ASHA Supervisor and other supervisors can visit some of the households which do not have trust and confidence on ASHAs technical competence and endorse their activities	X	√
	Some educated people do not consider ASHA's visit as important one; little trust on her technical competence	√	X	Beneficiaries of the service should motivate other members of the community to take the service, e.g. mothers in law motivate other elder women to follow ASHAs advices which did good to their families, women and children	X	√
	Poor respect of ASHAs based on the economic status of the families being visited during home visits	√	X	ASHAs should talk to both the mothers and grandmothers (mothers-in-law) together or separately for clear and consistent messaging; relationship between the two might influence communication and acceptance of the advice if given to only one of them	√	√
	Families/Clients which accepted immunization and institutional delivery advices of ASHAs many times get the honorarium late	√	X	Health department should particularly be cognizant of prompt release of the incentive money for the families/Clients for improving the credibility of ASHAs	√	X
**Educational**						
**Inadequate ASHA skills & training**	Inadequate and inappropriate of ASHA Training Program: limited technical competence and skill – training needs to better alignment with literacy and exposure level of the ASHAs	√	√	More the educational qualification, technical skill - more the respect for ASHAs; their credibility and respect shall increase in the community they serve and reduce discrimination	√	X
	ASHAs communication does not give trust and confidence to the families about the advice and referral to health facilities	√	√	Illiterate and less educated ASHAs should be changed	√	X
		√	√	A more knowledgeable person e.g. ANMs can accompany ASHAs occasionally; skilled ASHAs can also be used for peer training	√	X
				Emphasis on better and more comprehensive ASHA training as per the local realities; ASHAs must specially be skilled to identify red-flags/sickness in young infants, children and postpartum mothers	√	√
				Structured re-training for poor performing ASHAs before looking for their replacements	X	√
	Many ASHAs do not have HBNC plus training/ Training of new ASHAs is delayed	√	√	Ensure that all new recruits are trained and not inducted in to service straight away	√	√
**Organizational**						
Planning & Management of field activities	ASHAs did not plan their field work and home visitation; home visits were many times surprises to the mothers and other members of the family and therefore they did not get the attention and welcome ASHAs expected Lack of planning led to missing of home visits Lack of planning also affected all other public health activities of ASHAs No infrequently ASHAs re-prioritized their work based on directives from higher-ups for emerging health and non-health exigencies and missed home visits	√	√	ASHAs need to learn planning of home visitations; mothers in poor households are busy with too many activities including income generating activities and therefore some kind of prior information is necessary for the households. Mothers said if they knew the expected time of visits, they could telephone and call the ASHAs for the schedule visits.	√	√
	Home visitation tasks assigned to ASHAs are complex and are expected to impact community behavior toward health and wellness	√	√	The understanding of ASHAs should be clear and unambiguous so that she can impart the messages consistently and with confidence	√	√
	ASHAs are not able to do some home visits because the houses are located in outlying areas of villages – inhabited by low cast families	√	√	Involve Panchayat to encourage home visits by the ASHAs and not missing particularly in high risk populations Decrease the population size under ASHAs (if possible) in these situations	√	X
Supervision and mentoring	Lack of support from other frontline workers viz. AWWs, ANMs, ASHA Supervisor (Sahyognis) Involving and seeking community support	√	X	The department formally enlists the Institutions like VHND and VHNSC oversee that other frontline workers support ASHAs' work and facilitate activities in some of the households that are reluctant to participate and cooperate ASHAs' activities	√	√
				ASHA Supervisor should visit the village more frequently and accompany ASHAs at least for some home visits every month	√	X
				ASHA Supervisor should call a meeting of all ASHAs and bring the problem to the notice of elected village head (Sarpanch) (involvement of Sarpanch to increase social accountability)	X	√
	Lack of communication between ASHA and higher officials	X	√	ASHAs must bring her problems to the notice of the medical officer of the PHC/CHCs	X	√
**Economic**						
Incentive & availability of resources	ASHAs' families are not happy about ASHAs working for small amount of money, which is also not received in time	√	√	Consider higher incentives for ASHAs; ASHAs wanted fixed salaries and not honorarium; this factor was cited as an important reason for the noncooperation of ASHAs families	√	√
	ASHAs complained of not receiving their full due in time	√	√	If ASHAs get their dues completely and timely manner, their performance was likely to improve including home visitation; more support from families to travel at odd times with patients/pregnant women	√	√
	ASHAs cannot fill the claim forms/ delay in filling forms/poor maintenance of the records	√	X	ASHAs need regular handholding and help in filling the claim forms timely and correctly so that incentives are released fully and promptly	√	X
Travel & Communication Issues	No flexible funds available for ASHAs to hire vehicle for pregnant women/sick children in emergency/re-imbursements for telephone calls/SIM cards delayed	X	√	Money/call time for ASHA may be given in addition to SIM cards	X	√
**Physical**						
Transportation & Location Issues	ASHAs are unable to make home visits and travel with mothers and children to at odd hours; non-availability of transport facility to cross water channels between hamlets of a village	√	√	ASHAs' access to 108 in a manner that facilitates her mobility at all times and possibility of giving her flexible mobility funds	√	√
	At some places, the ASHAs are from villages different from their place of work; this is against the philosophy of ASHAs role as community worker. These ASHAs have frequent complaint of mobility and transport facilities	√	√	ASHA should be selected from the same village and not from a far off place/ Each village must have its own ASHA	X	√

Light Pink Color – Both the groups raised home visitation issues either as the challenge/barrier and or a potential solution.

Green – High caste group identified the issues either as the challenge/barrier and or a potential solution.

Blue – Low caste group identified the issues either as the challenge/barrier and or a potential solution.

ASHA, Accredited Social Health Activist; ANM, Auxiliary Nurse Midwife; AWW, Anganwadi Worker; MO, Medical officer.

**Figure 4:**
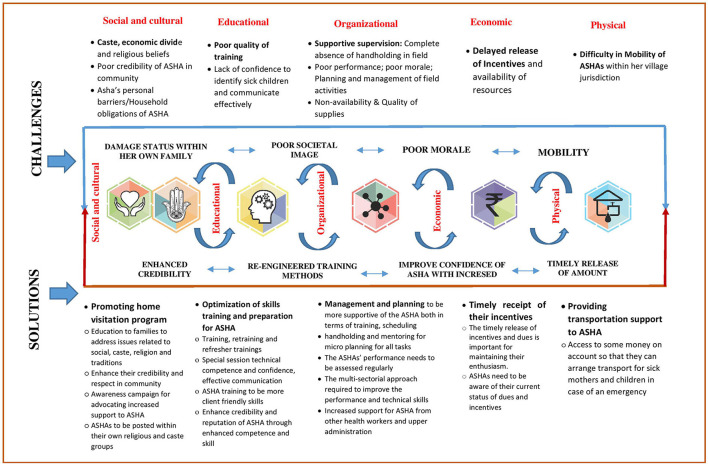
The challenges and solutions to improve ASHAs postnatal home visits.

The larger NPM group framed the recommendation at two levels: (i) administrative and organizational level; and (ii) community/family level. Task-oriented skill-building of ASHAs along with frequent re-orientation through supportive supervision and handholding was considered essential to improve ASHA performance, get her rightful place in her own family and the community, and even reduce caste-based prejudices. This will require re-engineering of the whole training curriculum in a manner that is aligned to the objectives of the HBNC and HBNC Plus programs. The ASHA was expected to prepare a micro-plan for her activities and communicate preventive, promotive, and curative knowledge to the families with confidence and conviction in their homes and community settings. Equally essential was to release the incentive money rightfully due to the ASHAs in a timely and regular manner. The health system was also asked by the nexus group to consider raising the incentive amount or making ASHAs regular salaried employees and consider the possibility of providing them with health insurance. The availability of flexible funds with ASHAs was considered useful for hiring vehicles at odd hours for the transport of sick children and pregnant women when ambulance services were not available. Another significant intervention proposed was increased male engagement in community meetings through village fora like VHND/VHSNC. These fora could help negotiate and reduce social discrimination and also strengthen social accountability (Figure 4).

## Discussion

The study adds to the existing knowledge that societal issues and personal challenges for ASHAs at their home and community fronts are among the more important barriers/challenges that prevented them from completing home visits and associated tasks with quality. The other reasons for incomplete age-appropriate home visits included gaps in the monitoring of ASHA's performance and mentoring system. Almost half of the ASHAs also accepted their lack of confidence in technical knowledge and communication skills. These factors shook community confidence in her capabilities and the importance given to her advice. The field-level staff including the ANMs, AWWs, block mobilizers, and ASHA supervisors strongly endorsed ASHAs viewpoint with particular emphasis on their social and domestic challenges, and importantly these were often overlooked. Mothers, even those never visited by ASHAs at home, felt that, ASHAs also lacked planning skills and consequently did not get the desired support from families for optimum performance.

The interconnectedness and complexity of the challenges in different sectors and aspects of human ecosystem have led to the recognition of the importance of transformative approaches in addressing and achieving sustainability (35). Nexus thinking and scenario planning have changed the way of doing things from linear to integrated and from monocentric to polycentric approaches (36). The novelty of the study is that it provides evidence of the interrelatedness of the multitude of barriers/challenges faced by the ASHAS along with solutions falling under the five nexus framework domains.

NPMs unambiguously recommended managing all the domains synchronously if the ASHA performance was to improve. The study data recommended interventions at the communication, organizational and educational levels to improve the quality of visits through enhanced skills and confidence of the ASHAs. This would necessitate reengineered training and follow up mentoring methods to enhance her credibility and respect in the community and abate to a certain extent the social and religious prejudices against the ASHAs. The social and family commitments of the ASHAs are important and complex challenges and need to be handled through engagement with the community at different levels e.g., Panchayati Raj members and the participation of men and elderly women in their families to help in negotiating ASHA's usefulness to the community with reduction of social discrimination; and enhancing her stature and respect in the community through reinforcement of her technical and communication skills. Incentives alone are insufficient to motivate the ASHAs for better performance as was done for HBNC Plus program. Timely release of due remuneration can be additional factors for motivating the ASHAs and soliciting their family support. The primary focus and responsibility of ASHAs can be better defined: if it remains maternal and child health activities, regular feedback, and monitoring will make them feel more responsible as well as accountable for different components of maternal and child health services.

The majority of the ASHAs in the current study perceived conflict between their domestic and professional responsibilities. Several previous studies have also shown that the personal needs and family commitments of community health workers interfered with their ability to make home visitations regularly and at times convenient to the beneficiaries (37–39). During the NPMs, the participants informed that the credibility and respect for the ASHAs particularly those from lower caste improved, and discrimination was less likely if they were technically competent, were not vague in their advice, and had good communication skills to impart messages with confidence and conviction.

The ASHAs have to navigate societal and cultural prejudices during their fieldwork (38) and household visits beyond their own “beradari” (caste/tribe/community group) were either not appreciated or trusted (40, 41). Another study reported maximum odds of getting ASHA's services if she belonged to a lower caste as she did not hesitate to visit a household from any community. In the present study, the reluctance to visit low caste households was more among the ASHAs from a higher caste (42). Communities are likely to trust their health workers if they are seen as part of the same community and background (43–46). The trust deficit compounded if ASHA's competence is suspected (47). Probandri et al. observed traditional practices related to the care of the new mother-child dyads also influenced the utilization of postpartum care advice and services. The social architecture further reinforced such practices when the mothers or mothers-in-law, members of the extended families, neighbors, and communities lived in the same or adjoining houses (48).

Mentoring and hand-holding emerged as important tools for the ASHAs' performance and quality of home visitations in several studies. Gilson et al. reported that high-quality supervision helped in the restoration and sustenance of trust of the community in the health system and its services (49). In a study from Ghana, 81% of the midwives had not been visited by their supervisors in time (50). Lack of supervision led to poor technical skills (51), stock-outs, and problems arising out of human resource deficit (52). The other reasons for the poor quality of home visitations were: inconsistent and non-reliable health messages, ‘always in hurry' attitude of the health worker during home visits, difficulty in contacting them when in need, non-provision of relevant health education materials, non-adherence to follow-up schedule (53, 54), and unwelcoming attitude toward the ASHAs by family members (55). Nammazi et al. found that knowledge of new born danger signs rapidly declined from 85.5 to 58.9% 1-year post-training and the main predictors of retention of community health worker knowledge were age (≥35 years) and post-primary level of education (56). The final NPM consultation observed that with the low level of education and social status in the community, current training formats were not suited for the ASHAs to acquire an adequate understanding of the complexity of the subject matter, and their subsequent translation in actual conditions. The group strongly recommended the need for re-engineering of training curricula, with a strong focus on technical skill building, developing self-assurance, and contextually understandable communication skills. Similar concerns and recommendations were reported in several other studies as well (57–60).

Complex interconnected challenges when addressed singly, at times may reduce one problem while exacerbating others. Studies have shown that tackling the challenges piecemeal has not helped to improve the ASHA's performance (61, 62). In a recent implementation study, the performance of ASHAs for home visitation and identification of sick young didn't improve significantly despite efforts by district health authorities to improve skill and technical competence (63). In a study by Creanga et al. (64) the mothers avoided accepting services from the ASHAs who couldn't deliver satisfactory quality postnatal home visitation despite having undergone training due to poor communication skills (64). Our study indicated that timely disbursement of incentives not only helped maintain their morale and motivation but also sent a positive message about their worth to their own families. Several studies have demonstrated a strong association between financial incentives and ASHAs performance (65, 66). Lewis and Bahety et al. also reported that low and delayed incentives were considered a major barrier to the service delivery by the ASHAs (67).

This study has some limitations. We applied NPF as a co-creation exercise involving a wide range of stakeholders and sectors for inductively developing a comprehensive understanding of the factors affecting home visitations by the ASHA, and the interrelatedness between barriers and potential solutions. We modified the broad principles of nexus planning for health system strengthening and improving the performance of frontline workers in resource constraint environments which also have deeply entrenched social, cultural, and other contextual factors. The rationale of giving importance and hearing to all the sub-group stakeholders/constituencies separately brought out several items and matters which required joint discussion to arrive at a mutually acceptable way forward. The challenges faced by ASHAs from forward and backward communities were different and had to be handled in a manner that required suggestions from both sides. The nexus approach guided us to identify and pursue synergies between health and non-health sectors i.e., Panchayati Raj [local self-government] recognizing their significant function and responsibilities to iron out some of the socio-cultural and economic biases operating at the village level. The feasibility and widespread applicability of NPF can be a concern. As mentioned above, successful NPF processes are dependent on identifying the sub-groups and sub-constituencies strategically for a series of group meetings/consultations. This will require deep insight into the local cultural, social, economic and, organizational milieu. Notwithstanding these limitations, the nexus framework did provide robust findings which can be triangulated with several published studies albeit done in a piecemeal manner from India and other low and middle- income countries (LMIC) settings. NPF has been successfully applied in environment, urban development, scrum methodology, water-energy-food (WEF) nexus approach in the MENA (Middle East and North Africa) countries, and many other areas for achieving sustainable development goals (SDGs) with sector-specific modifications (68). NPF offers a comprehensive structure for future researchers and implementation scientists to unravel the complexity and layers of health ecosystems and pursue efforts to improve the performance of community health workers. Research is also required to further refine NPF for wider application but its core philosophy should remain intact. Another limitation of the study can be the applicability of the findings from Raisen district to other parts of the state and country and other LMICs. The health indicators particularly related to tasks executed by ASHAs, Raisen falls in the lower one-third of the ranking of the Indian district (26). However, context and geography will continue to influence the relative significance of each of the five nexus domains.

In conclusion, the present study took a 360-degree view of the factors impacting the home visitation and related performance of the ASHAs and nexus planning groups emphatically recommended that each factor had an effect on or was dependent on the other factors and hence the remedies should include a more comprehensive approach rather trying for piecemeal solutions. The study showed that barriers and challenges of ASHAs can be classified under five broad domains. NPF stressed the imperative of approaching these barriers as interlinked challenges and resolution of any one component alone would not facilitate positive change. Future research and implementation agenda roadmap should include simultaneous interventions to improve the technical and communication skills to build the confidence of the ASHAs through reengineered training and follow-up mentoring methods; which is likely to enhance her credibility and respect in the community and abate a certain extent the social and religious prejudices against her. The social and family commitments of the ASHAs are important and complex challenges and need to be handled through engagement with the community at different levels. Another useful research agenda can be to determine the applicability of NPF in diverse settings and health system challenges.

## Data availability statement

The raw data supporting the conclusions of this article will be made available by the authors, without undue reservation.

## Ethics statement

The studies involving human participants were reviewed and approved by Ethics approval was obtained from the independent Ethics Committee of the INCLEN Trust International, New Delhi [DCGI reg no: ECR/109/Indt/DL/2014/RR-17]. Written informed consent to participate in this study was provided by the participants. Informant identity during the data analysis was kept anonymized. Verbal consent was taken from community representative and individual to participate in the nexus group meeting.

## Author contributions

NA designed the study. Data curation was done by VD, SJ, AP, RD, SC, KG, SB, BH, MD, RM, RS, and PS. Data analysis was done by NA, VD, SJ, AP, RD, and SC. NA, VD, and SJ prepared the first version of the manuscript. All authors provided comments on subsequent drafts and approved the final version of the manuscript.

## Funding

This work was supported by the Norway India Partnership Initiative (NIPI). [IND-16/0003 study of HBNC plus Project].

## Conflict of interest

The authors declare that the research was conducted in the absence of any commercial or financial relationships that could be construed as a potential conflict of interest.

## Publisher's note

All claims expressed in this article are solely those of the authors and do not necessarily represent those of their affiliated organizations, or those of the publisher, the editors and the reviewers. Any product that may be evaluated in this article, or claim that may be made by its manufacturer, is not guaranteed or endorsed by the publisher.

## Abbreviations

ASHA: Accredited Social Health Activists
ANM (Auxiliary nurse midwives
AWW: Aganwadi worker
BPM: Block program manager
BCM: Block community mobilizer
CDPO: Child Development Project Officer
DIO: District immunization officer
FGD: Focus group discussion
HBNC: Home-based newborn care
IFA: Iron and folic acid
IDI: In-depth-Interviews
LMIC: Low and middle income countries (LMIC)
MENA: Middle East and North Africa
NPF: Nexus Planning Framework
NIPI: Norwegian India Partnership Initiative
ORS: Oral rehydration solutions
SDG: Sustainable development goals
VHSNC: Village Health Sanitation &
Nutrition Committee
WEF: Water-energy-food.
